# Genome-wide association of echocardiographic dimensions, brachial artery endothelial function and treadmill exercise responses in the Framingham Heart Study

**DOI:** 10.1186/1471-2350-8-S1-S2

**Published:** 2007-09-19

**Authors:** Ramachandran S Vasan, Martin G Larson, Jayashri Aragam, Thomas J Wang, Gary F Mitchell, Sekar Kathiresan, Christopher Newton-Cheh, Joseph A Vita, Michelle J Keyes, Christopher J O'Donnell, Daniel Levy, Emelia J Benjamin

**Affiliations:** 1 The National Heart, Lung, and Blood Institute's Framingham Heart Study, Framingham, MA, USA; 2 Evans Department of Medicine and Whitaker Cardiovascular Institute, Boston University School of Medicine, Boston, MA, USA; 3 Department of Mathematics and Statistics, Boston University, Boston, MA, USA; 4 Veterans Administration Hospital, West Roxbury, MA, USA; 5 Cardiology Division, Massachusetts General Hospital, Harvard Medical School, Boston, MA, USA; 6 Cardiovascular Engineering, Inc., Waltham, MA, USA; 7 Program in Medical and Population Genetics, Broad Institute of Massachusetts Institute of Technology and Harvard University, Cambridge, MA, USA; 8 National Heart, Lung, and Blood Institute, National Institutes of Health, Bethesda, MD, USA

## Abstract

**Background:**

Echocardiographic left ventricular (LV) measurements, exercise responses to standardized treadmill test (ETT) and brachial artery (BA) vascular function are heritable traits that are associated with cardiovascular disease risk. We conducted a genome-wide association study (GWAS) in the community-based Framingham Heart Study.

**Methods:**

We estimated multivariable-adjusted residuals for quantitative echocardiography, ETT and BA function traits. Echocardiography residuals were averaged across 4 examinations and included LV mass, diastolic and systolic dimensions, wall thickness, fractional shortening, left atrial and aortic root size. ETT measures (single exam) included systolic blood pressure and heart rate responses during exercise stage 2, and at 3 minutes post-exercise. BA measures (single exam) included vessel diameter, flow-mediated dilation (FMD), and baseline and hyperemic flow responses. Generalized estimating equations (GEE), family-based association tests (FBAT) and variance-components linkage were used to relate multivariable-adjusted trait residuals to 70,987 SNPs (Human 100K GeneChip, Affymetrix) restricted to autosomal SNPs with minor allele frequency ≥0.10, genotype call rate ≥0.80, and Hardy-Weinberg equilibrium p ≥ 0.001.

**Results:**

We summarize results from 17 traits in up to 1238 related middle-aged to elderly men and women. Results of all association and linkage analyses are web-posted at http://ncbi.nlm.nih.gov/projects/gap/cgi-bin/study.cgi?id=phs000007. We confirmed modest-to-strong heritabilities (estimates 0.30–0.52) for several Echo, ETT and BA function traits. Overall, p < 10^-5 ^in either GEE or FBAT models were observed for 21 SNPs (nine for echocardiography, eleven for ETT and one for BA function). The top SNPs associated were (GEE results): LV diastolic dimension, rs1379659 (*SLIT2*, p = 1.17*10^-7^); LV systolic dimension, rs10504543 (*KCNB2*, p = 5.18*10^-6^); LV mass, rs10498091 (p = 5.68*10^-6^); Left atrial size, rs1935881 (*FAM5C*, p = 6.56*10^-6^); exercise heart rate, rs6847149 (*NOLA1*, p = 2.74*10^-6^); exercise systolic blood pressure, rs2553268 (*WRN*, p = 6.3*10^-6^); BA baseline flow, rs3814219 (*OBFC1*, 9.48*10^-7^), and FMD, rs4148686 (*CFTR*, p = 1.13*10^-5^). Several SNPs are reasonable biological candidates, with some being related to multiple traits suggesting pleiotropy. The peak LOD score was for LV mass (4.38; chromosome 5); the 1.5 LOD support interval included *NRG2*.

**Conclusion:**

In hypothesis-generating GWAS of echocardiography, ETT and BA vascular function in a moderate-sized community-based sample, we identified several SNPs that are candidates for replication attempts and we provide a web-based GWAS resource for the research community.

## Background

Cardiovascular disease (CVD) is a leading cause of morbidity and mortality in the United States [1]. It is increasingly recognized that CVD is a life-course disease, with overt events being antedated by subclinical cardiovascular target organ damage [2,3]. Current research indicates a fundamental role of left ventricular (LV) chamber size, wall thickness (LV remodeling) and mass (LVM) in the pathogenesis of high blood pressure [4,5], and clinical CVD [6,7], including stroke [8,9] and heart failure [10-12]. On a parallel note, exercise treadmill stress testing (ETT) is used routinely to evaluate patients with chest pain suggestive of ischemic etiology and for identifying individuals at intermediate pre-test probability of CVD who are more likely to develop clinical events [13]. Likewise, endothelial dysfunction, as assessed via brachial artery (BA) flow-mediated dilation (FMD), has emerged as a fundamental component of atherosclerosis and a precursor of overt CVD [14-16]. Thus, traits obtained via echocardiography (Echo), testing for BA endothelial function and ETT can serve as intermediate phenotypes in the pathway from standard risk factor to overt CVD. Such intermediate phenotypes have been studied extensively to characterize their clinical and genetic correlates, have been reported to be heritable traits [14,17-28], and have been linked to select genetic loci in several reports [29-31].

More recently, several investigators have proposed genome-wide association studies (GWAS) as a strategy to map causal genes with modest influences on traits associated with complex diseases such as CVD [32,33]. The availability of 100K genotype data on a subset of related Framingham Heart Study participants [34] provides a unique opportunity to conduct both genome-wide association and linkage analyses to explore the genetic underpinnings of LV remodeling, endothelial function and exercise performance in a community-based sample.

## Methods

The design and selection criteria of the Original Framingham Study [35] and the Offspring Study [36] have been described elsewhere. As detailed in the Overview [37], 1345 participants (1087 Offspring and 258 Original Cohort) underwent genotyping using the Affymetrix GeneChip Human Mapping 100K single nucleotide polymorphism (SNP) set [34]. Participants were eligible for the present investigation if they had available genotypes and the echocardiographic, vascular and ETT traits of interest (as defined below). The Institutional Review Board at Boston University Medical Center approved the study and all participants gave written informed consent (including for genetic research).

### Measurement of phenotypes

#### Echocardiography

All attendees underwent routine transthoracic two-dimensionally-guided M-mode echocardiography at the second (1979–1982), fourth (1987–1990), fifth (1991–1995) and sixth (1996–1998) Offspring cohort examinations. Echocardiographic equipment for image acquisition varied across these examinations: at examination cycle 2, a Hoffrel 201 ultrasound receiver (and Aerotech transducer) was used; at examinations 4 and 5, a Hewlett Packard (model 77020AC) ultrasound machine was used; at examination 6 images were acquired using a Sonos 1000 Hewlett-Packard machine. At all four examinations, however, measurements of LV internal dimension in diastole (LVDD) and systole (LVDS), the thicknesses of the posterior wall (PW) and interventricular septum (IVS), and the diameters of the aortic root (all measured at end-diastole) and left atrium (LA) at end-systole were obtained by using a 'leading edge' technique [38], averaging measurements in 3 cardiac cycles according to the American Society of Echocardiography guidelines. We calculated LV wall thickness (LVWT) as the sum of PW and IVS measurements. LV mass was calculated by using the formula 0.8[1.04(LVDD+IVS+PW)^3 ^- (LVDD)^3^] + 0.6 [39]. The reproducibility of Echo measurements was systematically assessed at the sixth examination [40].

#### ETT measures

At the second Offspring examination (1978–1981), all attendees underwent submaximal exercise test according to the standard Bruce protocol for up to five incremental 3-minute stages. The test was terminated (without a cool down period) when participants reached their target heart rate (85% age-predicted peak heart rate). Blood pressure measurements and electrocardiograms were recorded during exercise at the midpoint of each 3-minute exercise stage, and for each minute for up to 4 minutes into the recovery period.

#### BA endothelial function

As described previously [14], BA flow-mediated dilation (FMD; percent change in diameter from baseline; i.e. 100 * [hyperemic diameter at 1 minute - baseline diameter]/baseline diameter) and mean hyperemic flow velocity (cm/sec) were determined during the seventh clinical examination cycle (1998–2001). A Toshiba SSH-140A ultrasound system with a 7.5 MHz linear array transducer and commercially available software (Brachial Analyzer version 3.2.3, Medical Imaging Applications) were used. Investigators, blinded to participant clinical and genetic data, determined brachial artery diameter at baseline and 1 minute after reactive hyperemia induced by 5-minute forearm cuff occlusion. The coefficient of variation for baseline and hyperemic diameters were 0.5% and 0.7%, respectively.

Doppler flow was assessed at baseline and during reactive hyperemia using a 3.75 MHz carrier frequency and with correction for the insonation angle [41]. Mean baseline and hyperemic flow velocities were analyzed from digitized audio data using semiautomated signal averaging (Cardiovascular Engineering, Waltham, MA). Baseline and deflation flow measurements were reproducible on repeated analysis of 30 subjects with correlations of >0.98.

### Genotyping methods

The Overview [37] details the genotyping performed with the Affymetrix 100K SNP GeneChip http://gmed.bu.edu/about/genotyping.html[34] and with the Marshfield STR marker set at the Mammalian Genotyping Service http://research.marshfieldclinic.org/genetics.

### Statistical methods

We generated normalized sex-specific residuals adjusting for the following covariates: for the echocardiographic phenotypes, age, sex, height, weight, smoking, systolic and diastolic blood pressure, hypertension treatment; for ETT measures, age, sex, body mass index, baseline heart rate, diabetes, smoking, ratio of total to high-density lipoprotein cholesterol, and treatment for hypertension (additional adjustments for select variables is detailed in Table 1); for BA function, a set of 15 covariates previously reported [14] to be associated with endothelial function in our sample (see Table 1). Covariates were from the same exam as the phenotype measures. Next, we used residuals for the phenotypes of interest to test for potential association with 100K SNPs using additive family-based association tests (FBAT) and linear regression models with general estimating equations (GEE; additive genetic models) to account for correlation among related individuals from nuclear families, as detailed in the Overview [37]. We chose 70,987 SNPS for association analysis that met the following criteria: autosomal SNPs with genotypic call rate ≥80%, minor allele frequency ≥10%, Hardy-Weinberg equilibrium test p ≥0.001, and ≥10 informative families for FBAT. The choice of an 80% genotyping call rate threshold may appear unusually liberal. We chose this threshold to be more inclusive in terms of associations reported. Also, the algorithm for the genotype calls was the Dynamic Modeling algorithm, which is less precise than other algorithms that have been introduced more recently. The association analyses were complemented by linkage analyses that used variance components methods and a subset of 100K markers and Marshfield STRs; the selection of markers and methods for calculating identity-by-descent are also described in the Overview [37].

**Table 1:** Echocardiographic, exercise testing and brachial artery function traits analyzed in participants with 100K genotype data

Trait	Number of Traits*	Offspring Exam cycles	**Adjustment**^†^	Heritability
**A. Echocardiographic Traits Averaged Across 4 Examinations**				

LV mass (LVM)	10	2,4,5,6	- age- and sex- - multivariable-**	0.36
LV diastolic dimension (LVDD)	10	2,4,5,6		0.38
LV systolic dimension (LVDS)	10	2,4,5,6		0.30
LV wall thickness (LVWT)	10	2,4,5,6		0.41
LV fractional shortening (LVFS)	10	2,4,5,6		0.20
Left atrial diameter (LAD)	10	2,4,5,6		0.25
Aortic root diameter (AOR)	10	2,4,5,6		0.52

**B. Exercise Treadmill Test (ETT) Traits**				

Stage 2 Exercise systolic blood pressure (SBP)	2	2	- age- and sex- - multivariable-**	0.28
Stage 2 Exercise diastolic blood pressure (DBP)	2	2		0.22
Stage 2 Exercise heart rate	2	2		0.25
Post-exercise 3 minute recovery SBP	2	2		0.20
Post-exercise 3 minute recovery DBP	2	2		0.16
Post-exercise 3 minute recovery heart rate	2	2		0.40

**C. Brachial Artery (BA) Endothelial Function Traits**				

Baseline BA diameter	2	7	- age- and sex- - multivariable-**	0.25
Baseline BA flow velocity	2	7		0.32
BA flow-mediated dilation (FMD) percent	2	7		0.19
BA hyperemic flow velocity	2	7		0.06

BA = brachial artery. LV = left ventricular. SBP = systolic blood pressure. DBP = diastolic blood pressure.

* For Echo traits, the phenotypes listed include those based on averaged values across 4 examinations. Overall, the number of Echo phenotypes includes individual traits at each exam (× 2 for two levels of adjustment in models) plus the averaged traits across 4 exams (× 2 for two levels of adjustment in models) listed above. For ETT and BA traits, the number of individual traits includes traits at single exams (× 2 for two levels of adjustment in models).

**covariates in multivariable models include:

For Echo phenotypes: age, sex, height, weight, smoking, systolic blood pressure, diastolic blood pressure, hypertension treatment.

For ETT phenotypes: age, sex, BMI, diabetes, current smoking, baseline heart rate, hypertension treatment, total/HDL cholesterol. Additional adjustments were ETT phenotype-specific: Exercise SBP was also adjusted for systolic BP at rest; exercise DBP for diastolic BP at rest; exercise heart rate for heart rate at rest; Recovery SBP for systolic BP at rest, systolic BP during second stage of exercise, and peak systolic BP during exercise; Recovery DBP for diastolic BP at rest, diastolic BP during second stage of exercise, and peak diastolic BP during exercise; and recovery heart rate for heart rate at rest, during second stage of exercise, and peak heart rate during exercise.

For BA phenotypes: age, sex, mean arterial pressure, pulse pressure, heart rate, diabetes, body mass index, fasting blood glucose, prevalent cardiovascular disease, hormone replacement therapy use, walk test before and after BA test, Total/HDL cholesterol, smoking within 6 hrs of BA test, hypertension, lipid-lowering treatment use.

We used an unfiltered approach and report the top 25 SNPs associated with echocardiographic traits, ETT measures and BA phenotypes (15, 5 and 5 SNPs, respectively; relative proportions chosen empirically because of the larger number of echocardiographic traits analyzed) according to their degree of statistical significance (lowest p values) in GEE and FBAT models separately. For echocardiographic phenotypes, we analyzed the mean of values for traits averaged across the 4 examinations, as well as traits at individual examinations separately. In order to evaluate potential pleiotropic effects, we examined SNP associations across related sets of traits and listed the top 25 SNPs with the lowest geometric mean of p values for all echocardiographic traits (averaged across the four examinations), for all ETT measures and for all BA traits (15, 5 and 5 SNPs, respectively, for the 3 groups). Because we analyzed individual echocardiographic traits at each of the four examinations, we also listed the top SNP associations based on the geometric mean of p values for these individual echocardiographic traits across the four examinations. Thus, we use the term 'pleiotropic effects' to refer to whether there were SNPs that were associated with multiple traits within the 3 subgroups. Additionally, we examined associations of SNPs in or within 200 Kb of the start or terminus of six selected genes (*ACE, AGT, AGTR1, ADRB1, VEGF, NOS3*) that have been previously reported to be associated with Echo, ETT and BA function phenotypes [30,42-51]. We view these analyses as exploratory because the coverage of the Affymetrix 100K GeneChip for these genes was quite limited.

## Results

Table 1 lists the phenotypes analyzed from the three groups (Echo, ETT and BA endothelial function), the number of traits evaluated within each group, and the covariates included in regression models to create residuals. We observed moderate to high heritability of most of the traits evaluated (Table 1; estimates are multivariable-adjusted, for covariates noted above under methods, and listed in table footnote). Heritability estimates were 52% for aortic root dimension, 36–40% for LV mass, internal dimensions and LVWT, and 25% for LA size. Estimates for ETT measures varied from 41% for post-exercise recovery heart rate, 28% for exercise systolic blood pressure, and 16–25% for other phenotypes. For BA function, baseline flow velocity and vessel diameter were most heritable (32 and 25%, respectively) and hyperemic flow the least (6%), with intermediate values for FMD (19%).

Results of all association analyses and detailed linkage results are web-posted at http://ncbi.nlm.nih.gov/projects/gap/cgi-bin/study.cgi?id=phs000007, the database of genotype and phenotype public repository (dbGaP) at the National Center for Biotechnology Information. Overall, nine SNPs yielded a p value <10^-5 ^in either GEE or FBAT models for Echo traits. Eleven SNPs were associated with ETT traits with a p value <10^-5^, and one SNP yielded a p value below this threshold for BA function traits. A conservative Bonferroni correction for the number of statistical tests (0.05/1,000,000) yields an approximate threshold of genome-wide significance of 5*10^-8^.

Table 2A displays the 25 most significantly associated SNPs in GEE analyses (15 for echo phenotypes; 5 each for ETT and BA phenotypes; additive genetic models) sorted by p values along with the corresponding p value using FBAT. The top SNPs associated with averaged Echo traits included: rs1379659 and rs666088 (both in *SLIT2*) with LV diastolic dimension, rs1935881 (*FAM5C*) and rs10493389 (*PDE4B*) with LA diameter, and rs10504543 (*KCNB2*) with LV systolic dimension. The top SNPs associated with ETT traits included: rs6847149 (*NOLA1*) and rs2056387 (*RYR2*) with stage 2 exercise heart rate, and rs2553268 (*WRN*) with stage 2 exercise systolic blood pressure. The top 5 SNPs associated with BA traits included rs3814219 (*OBFC1*) and rs10515508 (*NRG2*) with BA baseline flow, and rs4148686 (*CFTR*) with FMD. Only one of these SNPs (*SLIT2*) had a p value < 10^-3 ^in FBAT.

**Table 2:** Top associations for Echo, ETT and BA function traits based on lowest p value for GEE test (2a), FBAT (2b), and Linkage (2c)*

2A. Top Associations based on lowest GEE p values						
**Trait**	**SNP**	**Chromosome**	**Physical position**	**GEE p-value**	**FBAT p-value**	**In/near Gene (within 60 kb)**

**I. Top 15 SNPs associated with Echocardiographic Traits (Averaged across exams)**						

LV diastolic dimension	rs1379659	4	20296952	1.17*10^-7^	0.001	*SLIT2*
LV fractional shortening	rs366676	6	88774485	2.44*10^-6^	0.059	*SPACA1*
LV diastolic dimension	rs666088	4	20171404	5.10*10^-6^	0.008	*SLIT2*
LV systolic dimension	rs10504543	8	73941196	5.18*10^-6^	0.247	*KCNB2*
Left atrial diameter	rs1935881	1	186798043	5.56*10^-6^	0.003	*FAM5C*
LV mass	rs10498091	2	221724949	5.68*10^-6^	0.162	
Left atrial diameter	rs10493389	1	66022886	6.56*10^-6^	0.119	*PDE4B*
LV diastolic dimension	rs4920799	5	84642284	6.84*10^-6^	0.041	
LV wall thickness	rs10519181	15	76076030	1.04*10^-5^	0.027	*TBC1D2B*
LV diastolic dimension	rs6104740	20	11284809	1.15*10^-5^	0.021	
LV diastolic dimension	rs2900208	12	11769731	1.20*10^-5^	0.021	*ETV6*
LV systolic dimension	rs3766377	1	157613632	1.25*10^-5^	0.032	*CD244*
LV diastolic dimension	rs10511762	9	25621130	1.29*10^-5^	0.002	*TUSC1*
LV mass	rs4936770	11	122434085	1.37*10^-5^	0.019	*HSPA8*
LV diastolic dimension	rs4485619	20	11251676	1.57*10^-5^	0.043	
						
**II. Top 5 SNPs associated with ETT Traits**						

Stage 2 Exercise heart rate	rs6847149	4	111157701	2.74*10^-6^	0.014	*NOLA1*
Stage 2 Exercise heart rate	rs2819770	1	234237045	3.53*10^-6^	0.010	*RYR2*
Post-exercise 3 minute recovery SBP	rs746463	11	109501154	4.88*10^-6^	0.564	
Stage 2 Exercise heart rate	rs2056387	1	234250153	5.17*10^-6^	0.002	*RYR2*
Stage 2 Exercise SBP	rs2553268	8	31055898	6.32*10^-6^	0.001	*WRN*
						
**III. Top 5 SNPs associated with BA endothelial function Traits**						

Baseline BA flow velocity	rs3814219	10	105637085	9.48*10^-7^	0.325	*OBFC1*
BA FMD percent	rs4148686	7	116728468	1.13*10^-5^	0.025	*CFTR*
Baseline BA flow velocity	rs1471639	14	33552277	1.26*10^-5^	0.001	
Baseline BA diameter	rs1045182	6	116705168	1.44*10^-5^	0.058	*TSPYL1*
Baseline BA flow velocity	rs10515508	5	139355254	1.71*10^-5^	0.016	*NRG2*

**2B. Top associations based on lowest FBAT p value**						

**Trait**	**SNP**	**Chromosome**	**Physical position**	**GEE p-value**	**FBAT p-value**	**In/near Gene (within 60 kb)**

**I. Top 15 SNPs associated with Echocardiographic Traits (Averaged across exams)**						

LV systolic dimension	rs1392284	3	114584100	0.139	6.39*10^-6^	*WDR52*
LV fractional shortening	rs10515040	17	48859319	0.211	1.29*10^-5^	
LV fractional shortening	rs9312006	3	8234491	0.002	1.64*10^-5^	
LV systolic dimension	rs10504591	8	76199715	0.046	1.95*10^-5^	
LV fractional shortening	rs448458	15	60591780	0.206	2.26*10^-5^	
LV diastolic dimension	rs580859	13	68030441	0.046	2.68*10^-5^	
LV systolic dimension	rs1959290	14	86208482	2.07*10^-5^	6.42*10^-5^	
Aortic root diameter	rs2468680	8	140590402	0.690	6.12*10^-5^	
LV systolic dimension	rs448458	15	60591780	0.019	5.60*10^-5^	
LV systolic dimension	rs2918268	5	148586949	0.574	4.82*10^-5^	*ABLIM3*
LV mass	rs965036	6	20099022	0.033	3.69*10^-5^	
Left atrial diameter	rs1701821	7	112544622	0.137	3.66*10^-5^	
Aortic root diameter	rs7544568	1	38308301	0.194	3.21*10^-5^	
LV fractional shortening	rs10504591	8	76199715	0.112	2.92*10^-5^	
LV diastolic dimension	rs1488745	3	1976802	0.141	2.72*10^-5^	
						
**II. Top 5 SNPs associated with ETT Traits**						

Post-exercise 3 minute recovery SBP	rs2016718	8	96813381	0.006	2.20*10^-7^	
Post-exercise 3 minute recovery heart rate	rs1029947	7	150713400	0.013	9.20*10^-7^	*PRKAG2*
Post-exercise 3 minute recovery heart rate	rs1029946	7	150713454	0.022	3.89*10^-6^	*PRKAG2*
Stage 2 Exercise heart rate	rs1958055	14	33254537	0.040	8.55*10^-6^	*NPAS3*
Post-exercise 3 minute recovery SBP	rs7828552	8	71862761	0.057	9.34*10^-6^	*XRG9*
						
**III. Top 5 SNPs associated with BA endothelial function Traits**						

BA hyperemic flow velocity	rs1859634	7	100758808	0.030	1.21*10^-5^	*AK124120*
BA FMD percent	rs1106494	14	62201734	0.003	1.61*10^-5^	*KCNH5*
BA hyperemic flow velocity	rs2389866	4	120872866	0.423	2.12*10^-5^	*PDE5A*
Baseline BA diameter	rs774227	9	91273496	0.002	2.34*10^-5^	*NFIL3*
Baseline BA diameter	rs10502887	18	43433881	0.002	2.79*10^-5^	
						
**2C. Magnitude and location of peak LOD scores ≥2.0 for Echo, ETT and BA function traits**						

**Trait**	**SNP or STR**	**Chromosome**	**Physical position**	**Maximum LOD score**	**LOD-1.5 interval**	**LOD+1.5 interval**

**I. Echocardiographic Traits (Averaged across examinations)**						

LV mass	rs10515509	5	139270238	4.37	133816612	150634674
Aortic root diameter	rs10513442	3	154474088	4.22	150552924	161813142
LV wall thickness	rs3813713	10	51241137	3.16	32818116	58684005
LV wall thickness	rs10511550	9	10638555	3.11	10220368	15384347
LV fractional shortening	AFM254ve1	3	198506417	2.78	195278503	199138789
LV wall thickness	rs2438085	2	105339005	2.59	103478258	106924695
Left atrial diameter	rs7327514	13	78334100	2.55	68655834	90511230
LV wall thickness	rs1719	15	83149176	2.45	64238853	86738287
LV mass	rs10489725	1	181172198	2.41	176249023	201595809
LV mass	rs1989051	8	129363300	2.38	127811630	133926399
LV wall thickness	GATA164B08	3	8560016	2.25	2183832	20875136
						
**II. ETT Traits**						

Stage 2 Exercise heart rate	rs190982	5	88259176	2.93	71236666	96112374
Stage 2 Exercise heart rate	GATA165C03	1	60383884	2.46	43070922	67006164
Stage 2 Exercise heart rate	rs7286558	22	20504737	2.43	15786453	25685518
Stage 2 Exercise heart rate	rs10483844	14	71902088	2.39	53240301	74937294
						
**III. BA Endothelial Function Traits**						

Baseline BA flow velocity	rs1425727	8	25642697	2.14	19459783	32261074
BA FMD percent	D21S11	21	19476134	2.13	10000969	27037800
Baseline BA flow velocity	rs3007456	9	42925816	2.12	36878975	76736108

*SNPs are ordered by GEE p values. dbSNP positions are from NCBI Build 35 (hg17).

Abbreviations as in Table 1. The number of informative families for FBAT analyses ranged from a minimum of 80 to a maximum of 210.

Table 2B lists the top 25 SNPs associated with phenotypes in FBAT along with corresponding p values in GEE models. Only one of these SNPs (rs1959290 associated with LV systolic dimension) had a p value < 10^-3 ^in GEE models. The top 5 SNPs associated with ETT traits included rs1029947 and rs1029946 (both in *PRKAG2*) with heart rate at 3 minutes of post-exercise recovery. The top 5 SNPs associated with BA traits included rs2389866 (*PDE5A*).

Table 2C lists the magnitude and the location of loci with LOD scores that exceeded 2.0. The peak LOD scores were: for Echo traits, 4.38 (chromosome 5) for LV mass and 4.23 (chromosome 3) for aortic root size; for ETT traits, 2.93 (chromosome 5), 2.46 (chromosome 1), and 2.43 (chromosome 22) for heart rate during stage 2 of exercise; for BA traits, 2.14 (chromosome 8) for baseline flow velocity.

Table 3 evaluates the potential pleiotropic effect of SNPs by evaluating the geometric mean of p values for associations across averaged echo, and single-exam ETT and BA traits, and by relating them to the individual Echo traits across 4 examinations. SNPs associated with 4 genes (*SLIT2, WDR72, UBE2L3 and KCNB2*) were related to individual echo traits across examinations, as well as associated with the averaged Echo traits. Likewise, *RYR2 *was associated with a low geometric p value when related to all ETT traits across the examination.

**Table 3:** Top associations ordered by geometric mean of GEE p-values across traits (3 groups) and across examinations (echo traits)

Trait	SNP	Chromosome	Physical position	GEE p-value	In/near Gene (within 60 kb)
**IA. Top 15 SNPs associated with Echocardiographic Traits across individual exams**					

LV diastolic dimension	rs4920799	5	84642284	0.001	
LV diastolic dimension	rs1379659	4	20296952	0.001	*SLIT2*
Aortic root diameter	rs26438	5	165221499	0.001	
LV mass	rs473664	15	51598287	0.0016	*WDR72*
Aortic root diameter	rs10488825	11	81688520	0.0017	
LV mass	rs10498091	2	221724949	0.0017	
LV diastolic dimension	rs10514431	16	76498035	0.0018	*KIAA1576*
LV diastolic dimension	rs10505599	8	133812159	0.0019	*FLJ33069*
LV systolic dimension	rs10504543	8	73941196	0.0019	*KCNB2*
LV diastolic dimension	rs2900208	12	11769731	0.0019	*ETV6*
LV mass	rs861857	22	20306894	0.0022	*UBE2L3*
LV systolic dimension	rs10501940	11	99440078	0.0023	*CNTN5*
LV systolic dimension	rs707025	2	155014031	0.0023	*GALNT13*
Aortic root diameter	rs1395204	18	4477509	0.0025	
LV diastolic dimension	rs10513272	9	116143601	0.0026	*PAPPA*

**IB. Top 15 SNPs associated with Averaged Echocardiographic Traits (across LV traits)**					

Across LV phenotypes	rs1379659	4	20296952	0.0007	*SLIT2*
	rs10498091	2	221724949	0.0009	
	rs861857	22	20306894	0.001	*UBE2L3*
	rs473664	15	51598287	0.0012	*WDR72*
	rs3766377	1	157613632	0.0016	*CD244*
	rs10504543	8	73941196	0.0017	*KCNB2*
	rs10518462	4	126562521	0.0018	
	rs1959289	14	86208319	0.0026	
	rs667269	3	175822307	0.0027	
	rs1959290	14	86208482	0.0027	
	rs1959291	14	86208525	0.0027	
	rs10485104	6	165966351	0.0030	*PDE10A*
	rs10491574	9	116701992	0.0030	*ASTN2*
	rs707025	2	155014031	0.0032	*GALNT13*
	rs525960	1	149310939	0.0033	*LCE5A*

**II. Top 5 SNPs associated with ETT Traits (across traits)**					

Across phenotypes	rs1560916	17	28973892	0.0101	*ACCN1*
	rs1432214	2	137712255	0.0106	
	rs10512056	9	76835140	0.0112	*LOC442425*
	rs2056387	1	234250153	0.0115	*RYR2*
	rs6560812	10	2363105	0.0132	

**III. Top 5 SNPs associated with BA endothelial Traits (across traits)**					

Across phenotypes	rs2912991	7	52684247	0.0029	
	rs10510677	3	36168070	0.007	
	rs10493052	1	33614778	0.007	*ZNF31*
	rs7155941	14	41681710	0.008	
	rs1954627	14	41689011	0.009	

dbSNP positions are from NCBI Build 35 (hg17).

Abbreviations as in Table 1.

Table 4A–C displays results of association of Echo, ETT and BA function traits with SNPs in proximity to 6 genes (within 200 Kb of the start or terminus) chosen from the published literature; as noted earlier, the Affymetrix 100K GeneChip does not cover SNPs within these genes adequately. We observed weak associations of several SNPs in proximity to the genes of interest and Echo, ETT and BA function traits, none of which had a p value < 10^-3^). It is noteworthy, though, that several SNPs in proximity to *ADRB1, AGT*, and *AGTR1 *were associated weakly (p value of 0.05 to 10^-3^) with several Echo, ETT and BA function phenotypes.

**Table 4:** Associations of traits with SNPs in or near (up to 200 kb away) 6 well-replicated genes in the published literature with a p-value < 0.05 in either FBAT or GEE.

4A. Associations of averaged echo traits					
**Candidate Gene**	**FHS 100K SNP**	**Physical Position**	**Trait**	**GEE p-value**	**FBAT p-value**

***ACE***	no SNP was associated with a p value < 0.05 for any LV trait studies				

***ADRB1***	rs10510001	115962372	LV diastolic dimension	0.004	0.0002
	rs10510001	115962372	LV systolic dimension	0.001	0.001
	rs10510000	115961985	LV diastolic dimension	0.052	0.001
	rs10510000	115961985	LV systolic dimension	0.059	0.001
	rs7902873	115884949	LV diastolic dimension	0.467	0.002
	rs10509999	115917269	LV diastolic dimension	0.136	0.003
	rs180940	115712401	LV wall thickness	0.003	0.072
	rs180934	115720720	LV wall thickness	0.005	0.090
	rs180935	115720249	LV wall thickness	0.005	0.160
	rs998334	115957002	LV diastolic dimension	0.289	0.005
	rs7902873	115884949	LV systolic dimension	0.249	0.006
	rs10509999	115917269	LV systolic dimension	0.036	0.008
	rs180935	115720249	LV mass	0.011	0.409
	rs180934	115720720	LV mass	0.015	0.411
	rs7902873	115884949	LV mass	0.646	0.016
	rs998334	115957002	LV systolic dimension	0.110	0.018
	rs10510001	115962372	LV fractional shortening	0.021	0.056
	rs10510000	115961985	LV fractional shortening	0.382	0.027
	rs180940	115712401	LV mass	0.029	0.303
	rs10510001	115962372	LV mass	0.393	0.040
	rs6585258	115739125	LV mass	0.047	0.481
	rs10510000	115961985	LV mass	0.335	0.048
***AGT***	rs10495300	227188497	Left atrial diameter	0.095	0.001
	rs2478518	227174605	LV fractional shortening	0.028	0.238
	rs1202585	227309949	LV wall thickness	0.030	0.028
	rs2180478	227295655	LV diastolic dimension	0.800	0.030
	rs758216	227005969	Aortic root diameter	0.031	0.341
	rs1202524	227257907	LV diastolic dimension	0.323	0.037
	rs2478518	227174605	LV systolic dimension	0.038	0.467
	rs731824	227329806	Left atrial diameter	0.043	0.399
	rs1202585	227309949	LV mass	0.044	0.117
***AGTR1***	rs1059502	150045008	Aortic root diameter	0.598	0.005
	rs1357424	149801524	LV fractional shortening	0.075	0.006
	rs1357424	149801524	LV systolic dimension	0.241	0.030
	rs1059502	150045008	LV diastolic dimension	0.142	0.030
	rs2331406	150048180	Aortic root diameter	0.589	0.032
	rs10513333	149786557	Left atrial diameter	0.437	0.039
***VEGF***	rs729761	43912549	LV fractional shortening	0.012	0.200
	rs729761	43912549	Left atrial diameter	0.573	0.018
	rs729761	43912549	LV systolic dimension	0.018	0.249
	rs2396083	43912786	LV fractional shortening	0.026	0.250
	rs2396083	43912786	Left atrial diameter	0.489	0.031
	rs2396083	43912786	LV systolic dimension	0.040	0.224

***NOS3***	No SNP was associated with a p value < 0.05 for any LV trait studied				

**4B. Associations of ETT traits**					

**Candidate Gene**	**FHS 100K SNP**	**Physical Position**	**Trait**	**GEE p-value**	**FBAT p-value**

***ACE***	rs10491167	58780430	Stage 2 Exercise systolic blood pressure	0.046	0.137
	rs10491168	58795195	Stage 2 Exercise systolic blood pressure	0.030	0.124
	rs721575	59136652	Post-exercise 3 minute recovery heart rate	0.024	0.025
***ADRB1***	rs2419857	115607366	Stage 2 Exercise heart rate	0.852	0.030
	rs4345919	115651155	Post-exercise 3 minute recovery heart rate	0.048	0.740
***AGT***	rs1752189	227000046	Post-exercise 3 minute recovery SBP	0.145	0.021
	rs758216	227005969	Post-exercise 3 minute recovery SBP	0.184	0.050
	rs10495298	227120049	Stage 2 Exercise systolic blood pressure	0.288	0.050
	rs2478516	227175387	Stage 2 Exercise systolic blood pressure	0.021	0.486
***AGTR1***	rs275678	149851039	Post-exercise 3 minute recovery SBP	0.216	0.049
	rs427832	149949061	Post-exercise 3 minute recovery SBP	0.014	0.171
	rs1949350	150089423	Stage 2 Exercise heart rate	0.010	0.335
***VEGF***	rs729761	43912549	Stage 2 Exercise heart rate	0.041	0.035
***NOS3***	rs2303928	150176978	Stage 2 Exercise heart rate	0.012	0.056

**4C. Associations of BA function traits**					

**Candidate Gene**	**FHS 100K SNP**	**Physical Position**	**Trait**	**GEE p-value**	**FBAT p-value**

***ACE***	no SNP was associated with a p value < 0.05				

***ADRB1***	rs180940	115712401	BA hyperemic flow velocity	0.581	0.015
	rs180935	115720249	BA hyperemic flow velocity	0.953	0.030
	rs180934	115720720	BA hyperemic flow velocity	0.662	0.029
	rs10509999	115917269	BA hyperemic flow velocity	0.001	0.137
	rs10510000	115961985	BA hyperemic flow velocity	0.007	0.014
	rs10510001	115962372	BA hyperemic flow velocity	0.017	0.239
***AGT***	rs758216	227005969	Baseline BA flow velocity	0.049	0.488
	rs1202585	227309949	Baseline BA diameter	0.002	0.080
	rs1202585	227309949	BA hyperemic flow velocity	0.049	0.080
***AGTR1***	rs1492090	149884751	BA hyperemic flow velocity	0.026	0.293
	rs427832	149949061	Baseline BA flow velocity	0.509	0.043
***VEGF***	rs833048	43762514	BA hyperemic flow velocity	0.860	0.036
	rs10498756	44046909	BA hyperemic flow velocity	0.312	0.008
***NOS3***	rs741067	149938103	Baseline BA diameter	0.043	0.024
	rs1006581	149949571	Baseline BA diameter	0.009	0.097
	rs2215564	150001612	BA hyperemic flow velocity	0.028	0.104

dbSNP positions are from NCBI Build 35 (hg17).

## Discussion

### Principal findings

We report results of GWAS of Echo, ETT and BA function traits in a moderate-size community-based sample using several complementary analytical approaches. Our principal findings are five-fold. First, we observed modest to strong evidence of heritability for several Echo, ETT and BA function traits, underscoring the contribution of additive genetic effects to interindividual variation in these traits. Our heritability findings confirm prior reports for some of the traits [18,20,22,23,27,28,52], including from our group [14,24]. Second, notwithstanding the modest-to-high heritability, none of the SNP-trait associations we observed achieved genome-wide significance (conservative Bonferroni correction p of 5*10^-8^). Therefore, any associations presented should be viewed as hypothesis-generating, with need for replication in additional samples. Third, our investigation highlights some of the challenges inherent in the interpretation of GWAS results. We did not observe any overlap between the top SNPs noted in GEE-based versus FBAT-based analyses, in part due to the inherent differences in the two analytical methods (see Overview for details [37]). Fourth, notwithstanding the lack of genome-wide statistical significance, our data do suggest several interesting biological candidates among the SNPs most strongly associated with different traits in the various analytical approaches (see discussion below). Fifth, we were quite limited in our ability to replicate findings for genetic variants previously associated with the traits that we investigated because specific coverage of such genetic variation in these candidates was limited in the Affymetrix 100K GeneChip. Therefore, the lack of replication of SNPs in proximity to 6 genes previously reported to be associated with Echo, ETT and BA traits should be interpreted with great caution. It is interesting that several weak associations (p between 0.05 and 10^-3^) were observed between traits in the three groups and SNPs in proximity to selected candidate genes evaluated (*ADRB1*, *AGT *and *AGTR1*).

### Potential biological candidates among observed associations

In our GWAS of Echo traits, a SNP in *SLIT2 *was associated with Echo LV diastolic dimension in several analyses. *SLIT2 *is an evolutionarily highly conserved gene that encodes a putative secreted protein, which contains conserved protein-protein interaction domains including leucine-rich repeats and epidermal growth factor-like motifs [53]. The gene has multiple effects but has been recently identified to have a novel role in vascular function by contributing to migratory mechanisms in vascular smooth muscle cells [54]. Likewise, the associations of LV mass with *HSPA8*, and of LA size with *PDE4B *are consistent with the key role of heat shock protein expression [55] and T-cell mediated immune responses [56], respectively, in myocardial hypertrophic responses to insults or hemodynamic overload.

Analyses of ETT traits provided some interesting results. The association of a SNP in *RYR2 *with exercise heart rate responses in multiple analyses is quite consistent with the fundamental role of the ryanodine receptor on the sarcoplasmic reticulum in calcium trafficking during cardiac muscle excitation-contraction coupling [57]. Furthermore, *RYR2 *has been implicated in exercise-induced polymorphic ventricular tachyarrhythmias [58]. Using FBAT, SNPs in *PRKAG2 *were associated with heart rate during the recovery period post-exercise. Mutations in *PRKAG2*, an enzyme that modulates glucose uptake and glycolysis [59], are associated with glycogen-filled vacuoles in cardiomyocytes. The phenotypic manifestations include cardiac hypertrophy, ventricular pre-excitation and conduction system disturbances, encompassed together in the Wolff-Parkinson-White syndrome [60].

Genetic linkage analyses of ETT traits identified peaks on chromosomes 5 and 22 for exercise heart rate. The 1.5 LOD support intervals for these peaks included *MEF2C *and *MAPK1*, respectively. *MEF2C *is a critical regulator of cardiac morphogenesis [61]. Additionally, overexpression of *MEF2C *in experimental studies is associated with disturbances in extracellular matrix remodeling, ion handling, and metabolism of cardiomyocytes [62]. The peak on *MAPK1 *is of interest because a recent investigations highlighted the role of MAPK signaling in mediating the responses of skeletal muscles to exercise training [63].

A SNP in *NRG2 *was associated with BA flow velocity at rest, and also was in proximity to the top LOD peak for LV mass, raising the possibility of pleiotropic effects of this gene on ventricular and vascular remodeling and function. *NRG2*, which encodes neuregulin-2, is a member of the epidermal growth factor (EGF) family and binds to ErbB receptors. ErbB signaling has been implicated in angiogenesis and endothelial cell proliferation [64]. Of interest, a SNP in *CFTR *was associated with FMD. It is noteworthy that *CFTR *is expressed in vascular smooth muscle cells and activation of *CFTR *chloride channels regulates contraction and relaxation of smooth muscle cells; disruption of the *CFTR *gene prevents cAMP-dependent vasorelaxation in experimental studies [65]. *CFTR *is also expressed in endothelial cells where it functions as a cyclic nucleotide-regulated chloride channel [66]. Of interest, a SNP in *PDE5A *was associated with BA hyperemic flow velocity in FBAT analyses. Phosphodiesterase 5 (PDE5) hydrolyzes cyclic guanosine monophosphate (cGMP) and cyclic adenosine monophosphate (cAMP), is widely expressed in the vasculature, and is best known as the target of sildenafil, a drug used to treat erectile dysfunction [67]. PDE5 degrades cGMP in smooth muscle cells so as to maintain the contracted state of blood vessels [67]. *PDE5A *may also play a critical role in the growth promoting effects of Angiotensin II on vascular smooth muscle cells [68].

### Strengths and limitations

The moderate-sized community-based sample, availability of longitudinal Echo measurements, routine ascertainment of standardized and reproducibly-measured traits, and evaluation of multiple complementary analytical methods including assessment of potential pleiotropic genetic effects strengthen our investigation. By web-posting unfiltered aggregate data we provide a resource for the scientific community to conduct in silico replication. Nonetheless, several limitations must be emphasized. As noted previously, the lack of genome-wide significance for any association observed given the extent of multiple statistical testing does not exclude a potential role of genetic influences on the traits studied. We had limited statistical power to detect modest genetic effects, given the sample size and the extent of multiple testing. As detailed in the Overview paper [37], for a conservative alpha level such as 10^-8^, we have more than 90% power to detect an association with a SNP explaining 4% or more total phenotypic variation when 80% or more individuals are phenotyped. We also had limited ability to replicate previously reported findings, in view of the partial coverage of genetic variation in select candidates with the Affymetrix 100K gene chip. Additionally, genetic variants may influence phenotypes in a context-specific manner [69], being modulated by environmental influences. For instance, the associations of *ACE *and *AGTR2 *with LV mass were reported to vary according to dietary salt intake in one investigation [48]. We did not undertake an investigation of gene-environmental interactions in the present study. Likewise, some of the moderately strong associations may represent false-positive results, notwithstanding the evidence suggesting that some of the associated SNPs may be reasonable biological candidates. We averaged echocardiographic traits across multiple examinations, with a view to characterizing the phenotype better over a period of time using several observations. Such a strategy could limit regression dilution bias, if the examinations are repeated over a short period of time. In our study, however, these examinations spanned a time period of twenty years, and the examinations used different echocardiographic equipment that may introduce misclassification. Further, such averaging assumes that similar sets of genes and environmental factors influence traits over a wide age range. Such an assumption may not be true, i.e., age-dependent gene effects may be masked by averaging of observations across ages in participants. Lastly, our sample was white and of European descent. The generalizability of our findings to other ethnicities is unknown.

## Conclusion

In hypothesis-generating GWAS of Echo, ETT response and BA vascular function in a moderate size community-based sample, we identified several SNPs that are potential candidates for replication. Overall, our investigation provides a scientific framework for analyzing and interpreting GWAS of phenotypes fundamental to our understanding of cardiac and vascular remodeling and hemodynamic responses to exercise testing. We expect the Framingham 100K SNP data to serve as a valuable scientific resource by virtue of the web-posting of unfiltered aggregate data

## Abbreviations

BA = brachial artery. CVD = cardiovascular disease. ETT = exercise treadmill test. FBAT = family-based association test. FHS = Framingham Heart Study. FMD = flow-mediated dilation. GEE = generalized estimating equations. GWAS = genome-wide association. IVS = interventricular septum. LA = left atrium. LOD = logarithm of odds. LV = left ventricular. LVM = LV mass. LVWT = LV wall thickness. LVDD = LV diastolic diameter. LVSD = LV systolic diameter. PW = posterior wall. SNP= single nucleotide polymorphism.

## Competing interests

None of the authors have a competing interest relevant to the subject of this manuscript. GFM is the owner of Cardiovascular Engineering, Inc, a company that designs and manufactures devices that measure vascular stiffness. The company uses these devices in clinical trials that evaluate the effects of diseases and interventions on vascular stiffness.

## Authors' contributions

RSV conceived the study design, planned the analyses, drafted and critically revised the manuscript. MGL planned the study, assisted in securing funding for BA function, planned and conducted the analyses, and critically revised the manuscript. JA critically revised the manuscript. TJW contributed to the analysis and interpretation of data, revising of the manuscript for important intellectual content. GFM assisted in obtaining funding for BA function measures, contributed to data acquisition, and critically revised the manuscript. SK and CNC participated in the study design, interpretation of data, and reviewed the manuscript. JAV assisted in securing funding for BA function measures and revising the manuscript. MJK contributed to collecting the BA function data base and reviewing the manuscript. COD and DL participated in the study conception and design, interpretation of data and reviewed the manuscript. EJB provided critical input designing the study, securing funding for BA function and echo measures, acquiring measurements, planning the analyses and critically revising the manuscript.
